# First longitudinal study of seal-feeding killer whales (*Orcinus orca*) in Norwegian coastal waters

**DOI:** 10.1371/journal.pone.0180099

**Published:** 2017-06-30

**Authors:** Eve Jourdain, Dag Vongraven, Anna Bisther, Richard Karoliussen

**Affiliations:** 1 Norwegian Orca Survey, Andenes, Norway; 2 Norwegian Polar Institute, Tromsø, Norway; 3 Reportagebörsen, Gothenburg, Sweden; Centre National de la Recherche Scientifique, FRANCE

## Abstract

Killer whales (*Orcinus orca*) have been documented preying on either fish or marine mammals in several regions, suggesting that this odontocete species has the ability to specialize on different types of prey. Off Norway, killer whales have been shown to rely on the Atlantic herring (*Clupea harengus*) as a main prey resource. Infrequent observations have revealed seals as an additional component of their diet, yet the extent of predation on marine mammals has remained largely unknown. Here, we present the findings of 29 years of photographic and observational data on seal-feeding killer whale groups identified in Norwegian coastal waters. Four groups have been observed preying and feeding on seals over several years, taking both harbor (*Phoca vitulina*) and grey (*Halichoerus grypus*) seals. These stable groups are shown to adopt small group sizes, were typically observed in near-shore areas and were not encountered on herring wintering grounds. Behavioral and social traits adopted by these groups are similar to those of pinniped-feeding killer whales from other regions. The potential ecological reasons and the extent of such prey specializations are discussed.

## Introduction

Killer whales (*Orcinus orca*) are cosmopolitan apex predators, preying on over 140 prey species including bony and cartilaginous fish, squids, mammals, reptiles and birds throughout their cosmopolite range [1, 2]. Despite a broad diet, some local populations feed on a narrow range of prey, adapting feeding strategies to prey type and availability [3–6].

In coastal waters of the eastern North Pacific, the most extensive studies have been focused on two sympatric ecotypes of killer whales, differing in morphology, pigmentation, acoustics, social behavior, group size, diet, movement patterns and genetics [4, 7–13]. The so-called resident type appears to be exclusively fish-feeding, preferentially preying on salmon species, whereas the transient type preys exclusively on marine-mammals [4, 10, 12, 14].

More recently, similar ecological specializations have been identified in other regions; off southeastern Alaska [5], Russian Far East [15] and in Antarctica [6, 16, 17]. Interestingly, killer whales thought to largely or exclusively specialize on pinniped-prey seem to adopt identical behavioral and social adaptations as described for transient killer whales [18–20]. A convergence of plastic traits has been suggested as an adaptation to the type of prey being hunted [21–23].

In the North Atlantic, similar variations in killer whale feeding behavior have been suggested but never sufficiently documented, and a possible ecotype delineation is less clear [24, 25]. Based on analyses of nitrogen stable isotopes and tooth wear in samples from museum and stranded specimen, Foote and colleagues [26] suggested the presence of a generalist Type 1 and a marine-mammal specialist Type 2, the two being partly sympatric in some overlapping regions of their respective ranges. Killer whales ranging off Norway, Iceland and in the North Sea were proposed to be the Type 1, having the Atlantic herring (*Clupea harengus*) as a main prey resource but with individual and/or group variations in the proportions of prey items consumed [26]. An ecological gradient was suggested within this Type 1, with some groups persistently feeding on higher trophic level prey.

Off Norway, research conducted over the past three decades has largely focused on areas where killer whales congregate seasonally in large numbers owing to abundance of the Norwegian Spring Spawning (NSS) stock of the Atlantic herring [27, 28]. Seasonal movement pattern, site fidelity of identified individuals as well as complex coordinated feeding behaviors highlighted killer whales as herring specialists [27–29]. Sporadic predation observations also suggested additional types of prey including seals and harbor porpoise that support individuals feeding at higher trophic levels [24, 30, 31]. However, because these opportunistic observations often lack identification of individuals, the potential ecological specializations of marine mammal eating killer whales in Norwegian waters has remained largely undocumented.

In this study, we document identified killer whales that have been persistently feeding on seals for a minimum of 30 years in Norwegian coastal waters. To determine if these whales displayed similar adaptations to pinniped-feeding killer whales in other regions, and as a first step towards assessing the scope of prey specialization, we looked into specific occurrence patterns, foraging behavior and social structure. Using photo-identification and predation data collected from 1988 through 2016, the specific aims of the study were to 1) highlight apparent prey specialization of seal-eating killer whales and 2) provide the first substantial baseline information about killer whale predation on pinnipeds in Norwegian waters, while contributing data on behavioral characteristics of seal-feeding killer whales from a newly investigated region.

## Materials and methods

### Data collection

**Fig 1:**
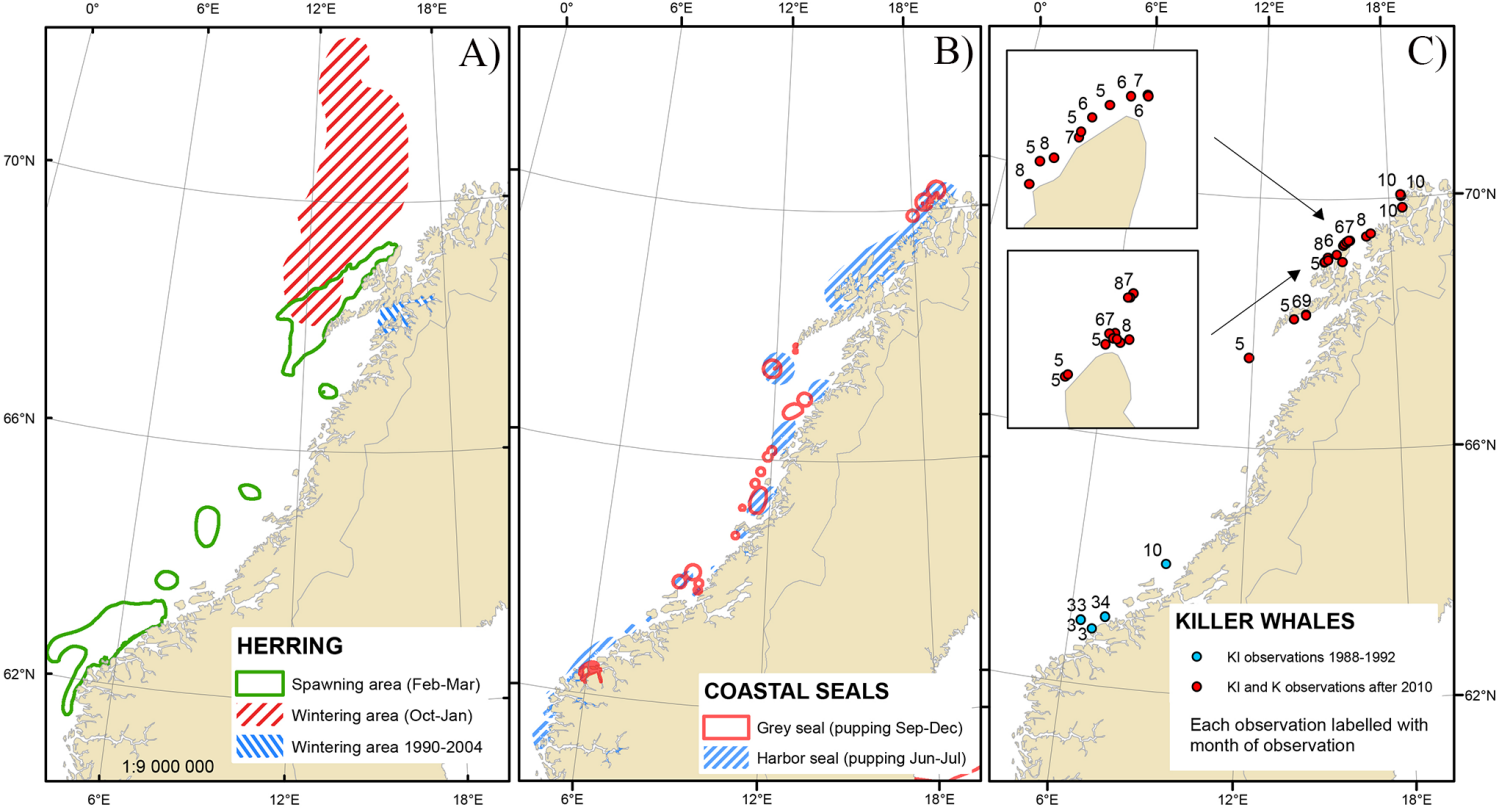
Important prey areas and observations of seal-feeding killer whale groups. The left panel (A) shows seasonal herring grounds since 1990 while the middle panel (B) shows areas of abundance of coastal seals since 1990 [32]. The right panel (C) shows locations of K and KI whales sightings since 1988. (The map was made in ESRI ArcGIS software [33] using public domain map data @ naturalearthdata.com).

The data set used for this study consisted of records of photo-identified individual killer whales and observations of predation events from different periods and various areas (Fig 1) as follows:

1. Long-term killer whale study initiated in 2013 in northern Norway [34]:
  Records of predation events involving identified killer whales were collected year round off Andenes, northern Norway (approximately 69°19'N, 16°08'E; Fig 1), as part of a study on the foraging ecology of killer whales. Field efforts began after a sighting report from a network of local people including fishermen, all-seasons whale-watching companies and local inhabitants. Data collection was also conducted seasonally further north, off Troms (69°50’N, 18°30’E), in October-November 2014, 2015 and 2016. On each encounter, information including date, time, location, group size estimate and group composition was logged. Using a DSLR camera equipped with a telephoto lens, individual killer whales were systematically photographed throughout the encounters. On a few occasions, highly distinctive individuals were identified from direct observations in the field. Behavior and predation events were recorded using *Ad libitum* sampling methods [35]. Surfacing whales displaying speed bursts, sudden changes in direction and circling behaviors often indicated ongoing predation incidents that were documented with photographs and/or video recordings. Whenever possible, prey species was visually identified on the field or through photographs. When the whales had finished feeding and moved on, the feeding spot was approached and prey remains collected using a fine-mesh dip net to confirm prey consumption. Encounters ended when weather, light and/or sea conditions became too poor to continue or if visual contact with the whales was lost.
2. Photo-identification study based on central Norwegian coast 1987–1993 [24]:
  A photo-identification study was initiated in 1987 in areas covering the herring spawning ground that was mainly located off the Møre coast, and approximately 100 nm of coastline north from this area (between latitudes 62°45’N–64°05’N and longitudes 6°–9°15’E, Fig 1). From 1987 through 1993, the collecting of ID photographs was opportunistic and conducted from November through March, resulting in a catalogue of 605 individuals [24]. All photographs were taken with analogue cameras, fitted with 300 mm telephoto lenses and predation events were recorded *Ad libitum* [35]. Information including date, time, location and group size estimate was noted during each encounter. Additional fieldwork was conducted around harbor and grey seal pupping colonies in June and September-October, respectively. Field studies were conducted in Møre areas in the years 1987–1993 sporadically during the months November through March. In the period 1990–1993 field studies during October through December were carried out in the Tysfjord area (68°16'N, 15°52'E) for four consecutive years.
3. Photo-identification study based in northern Norway 1986–2003 [36]:
  A catalogue of 585 individual killer whales photographically identified in northern Norway, as part of a long-term killer whale study, was available for review. This catalogue consisted of photographs taken in northern Norway in the former NSS herring wintering ground in Tysfjord-Ofotfjord-Vestfjord from 1986 to 2003 (work of Similä and colleagues mentioned in [36]).
4. Opportunistic imagery from other sources:
  One video containing unidentifiable whales preying on seals was collected from a wildlife photographer in 1988 at Orskjera, off the Møre coast (63°7’N, 7°15’E). Substantial amounts of high resolution opportunistic photographs, taken from 2010 through 2016 and accompanied by metadata on date and location, were also provided by wildlife photographers. Photographs were taken in all seasons in coastal waters between latitudes 62° and 72° (Fig 1). Six videos showing killer whales hunting seal prey, including two accompanied by high resolution photographs, were also available for review.

### Data analysis

All accounts of killer whales preying on seals were recorded, including both successful and failed predation events. Following photo-identification protocols developed by Bigg [7], individual killer whales specifically involved in these predation events were identified using the shape, pigmentation and scarring patterns of the dorsal fin and saddle patch. Photographs of both left and right sides were used for individual identification. Best photographs for each individual in each encounter was selected and rated for quality (Q), ranging from 0 (lowest) to 2 (highest) based on sharpness, contrast and angle of the dorsal fin in relation to the camera. All further sightings of individual killer whales identified as seal-feeding were recorded, including information on date and location. Resulting sighting histories, including only identifications of quality Q1 and Q2, were further used to map temporal and spatial occurrence patterns.

A group of whales was defined as all individual killer whales visible within the range of the observer and displaying coordinated activities (as per [11, 22, 37]). To assess group size, only encounters for which all individuals were believed to be identified were used. Group sizes adopted by seal-feeding *vs* fish-feeding killer whales were compared using a Mann-Whitney-Wilcoxon test performed in R [38].

Association patterns between individuals and persistence of groups were analyzed utilizing SOCPROG 2.6, a software specifically designed to study social organization of animal populations [39]. Association indices were calculated for all pairs of individuals using the Simple-Ratio Index, with association defined as presence in the same group within a sampling period (i.e an encounter on a certain date) and with all group members presumed identified [40]. From opportunistic photographs, group membership could be determined by looking at individuals captured within frames, or by pooling frames within certain time intervals [21]. Unreliably marked calves were excluded from these analyses and only Q2 identifications were accounted for. In order to reduce bias associated with small sample sizes, restrictions were also set to account exclusively for individuals observed in at least four sampling periods (as per [41]). The resulting association matrix was summarized in a sociogram with nodes representing individuals and edges indicating incidence and strength of relationship between each pair of whales [42]. Permuting associations within samples enabled testing for potential preferred associations among individuals [43, 44]. If the standard deviation (SD) calculated on the pairwise associations was significantly greater for the real association indices than the random data set, the null hypothesis of individuals associating at random was rejected. To further investigate temporal patterns in detected associations, the lagged association rate was calculated [45, 46]. This analysis calculates the probability for two animals to be associated again after some time, given that they have been associated before. The null association rate, which is the expected value of the lagged association rate if individuals associate at random, was plotted for comparison.

## Results

A total of 23 predation events of killer whales on seal prey in Norwegian coastal waters was documented from 15,000 photographs taken over 300 days of research effort between March 1988 and June 1992 [24], and 158,000 photographs, six videos and field notes taken over 468 days between July 2010 and December 2016. Nineteen of these predation events resulted in kills and consumption of the prey, and four were unsuccessful chases. Positive identification of individual killer whales involved was possible for 14 of these 23 predation events, and these whales were further re-sighted on 23 encounters, with no observed predation. Both young and adult individuals of grey (*Halichoerus grypus*) and harbor seals (*Phoca vitulina*) have been observed as part of the whales’ chosen pinniped prey.

### Identification of seal-eating killer whales

**Fig 2:**
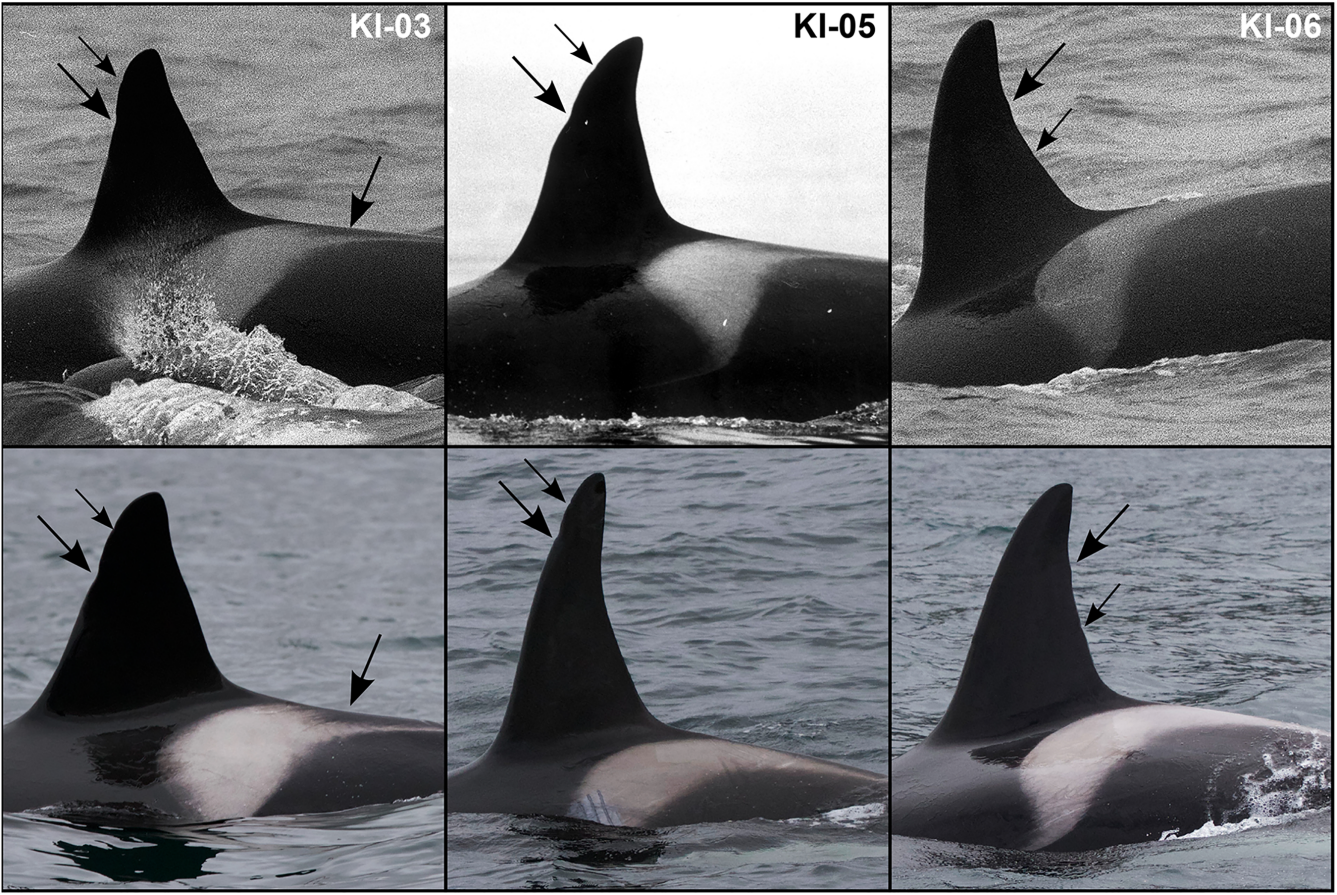
Photographs of seal-feeding killer whales KI-03, KI-05 and KI-06. Individuals in upper photographs, observed off central coast of Norway in 1988, match individuals visible on the lower row of photographs, taken off Andenes in June 2015.

Five killer whales, including so-called KI-03, KI-05 and KI-06, were first identified chasing harbor seals in March 1988 off the central coast of Norway. Three more individuals were identified in this group while foraging in a grey seal pupping colony in October 1990, before being identified as part of a larger group of 11 whales in March 1991. KI-03, KI-05 and KI-06 were re-sighted on several occasions between May 2011 and August 2016, in northern Norway (Fig 2). To ensure consistence with the longitudinal study, all individual whales encountered with KI-03, KI-05 and KI-06 on at least one occasion between 2010 and 2016 were given similar alphanumeric codes and considered as members of the KI group.

**Fig 3:**
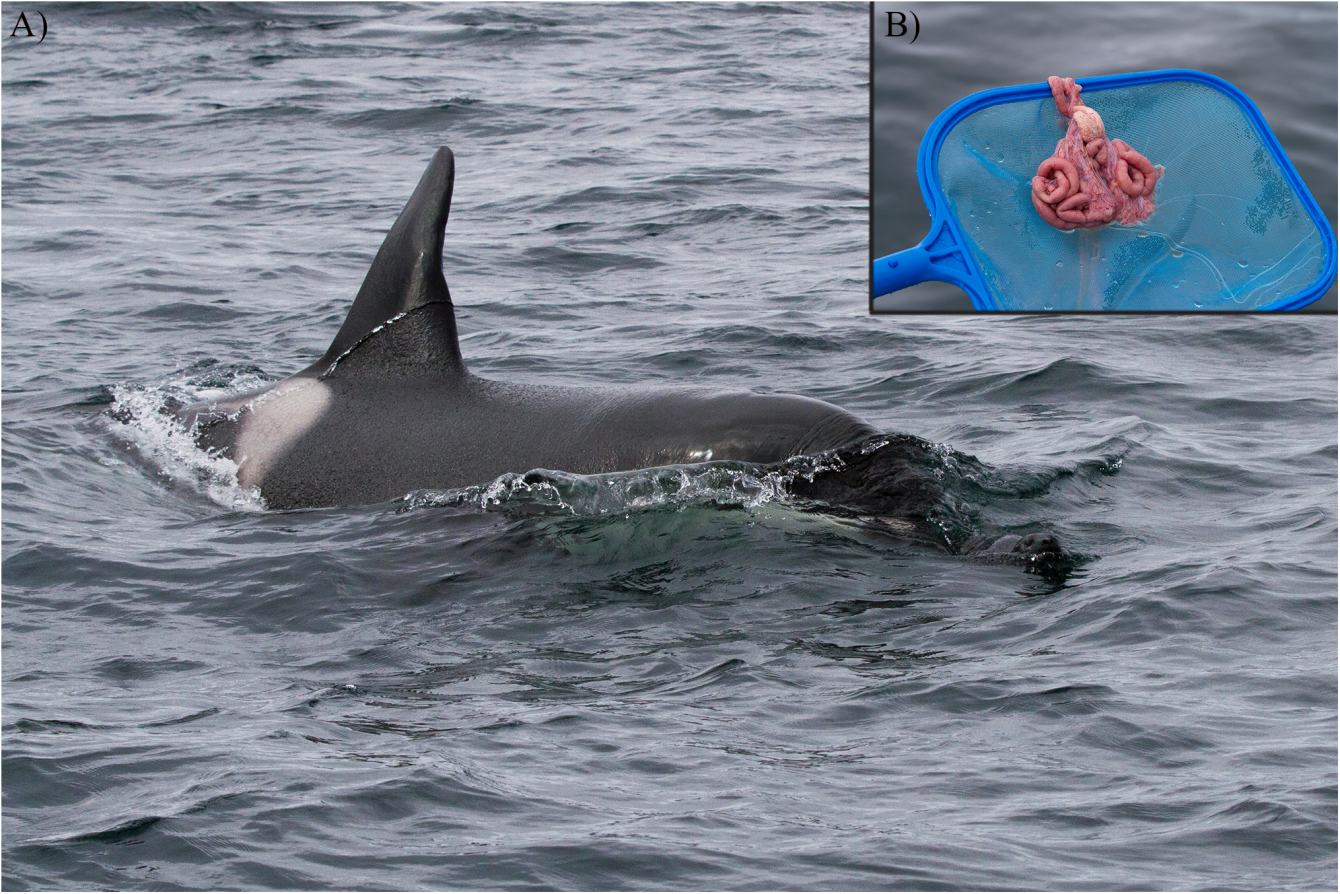
Photograph of KI-03 preying upon a harbor seal pup in June 2015. The photograph (A) illustrates the predation observation and the inset (B) confirms effective prey consumption.

KI whales were observed chasing or preying on pinnipeds on eight occasions (Fig 3). They were also observed twice feeding on herring at herring spawning grounds in 1991 off the Møre region, in association with other known herring-feeding killer whales, before travelling to a seal haul-out where they were observed for several hours unsuccessfully chasing harbor seals.

First identified in July 2010, individuals K1, K2, K3, K4 and K5, hereafter referred to as the K group, constitute the second assemblage of killer whales identified as seal-feeding.

K whales were observed chasing or preying on seals on six occasions and were never observed feeding on fish during the course of this study.

Predation observations for KI and K whales are summarized in Table 1. There was no evidence for interaction between KI and K whales in this study.

**Table 1:** Summary of recorded predation events on seals where involved individual killer whales could be identified.

Date	Location	Predation type	Minimum whales identified	Seal species	Evidence
27 Mar 1988	Orskjera, Møre	Chase	KI03, KI05, KI06	Harbor seal	Video
15 Oct 1990	Froan, Trøndelag	Blubber feeding	KI03	Undetermined	Photographs
10 Mar 1991	Ona, Møre	Chase	KI03, KI05, KI06	Harbor seal	Photographs
27 Jun 2013	Stø, Nordland	Kill	KI02, KI05, KI06, KI08, KI09, KI10	Undetermined	Photographs
15 Jul 2013	Andenes, Nordland	Kill	K1, K2, K3, K4, K5	Harbor seal	Photographs
27 Jul 2013	Andenes, Nordland	Kill	KI01, KI03, KI07	Harbour seal	Photographs and video
30 May 2014	Andenes, Nordland	Kill	K1	Undetermined	Photographs
31 May 2014	Andenes, Nordland	Kill	K1, K2, K3, K4, K5	Undetermined	Photographs and video
14 Sept 2014	Vestfjord, Nordland	Chase	K1, K2, K3, K4, K5	Harbour seal	Video
27 Jun 2015	Andenes, Nordland	Kill (3)	KI01, KI02, KI03, KI04, KI05, KI06, KI07, KI08, KI09, KI10	Harbour seal (1), grey seal (1), undetermined (1)	Photographs
19 Aug 2016	Senja, Troms	Kill (2)	K1, K2, K3, K4, K5	Harbour seal (1) and undetermined (1)	Photographs and video

For encounters that occurred in the period 1988–1991, only identifications of whales re-sighted after 2010 are indicated.

### Occurrence pattern

**Fig 4:**
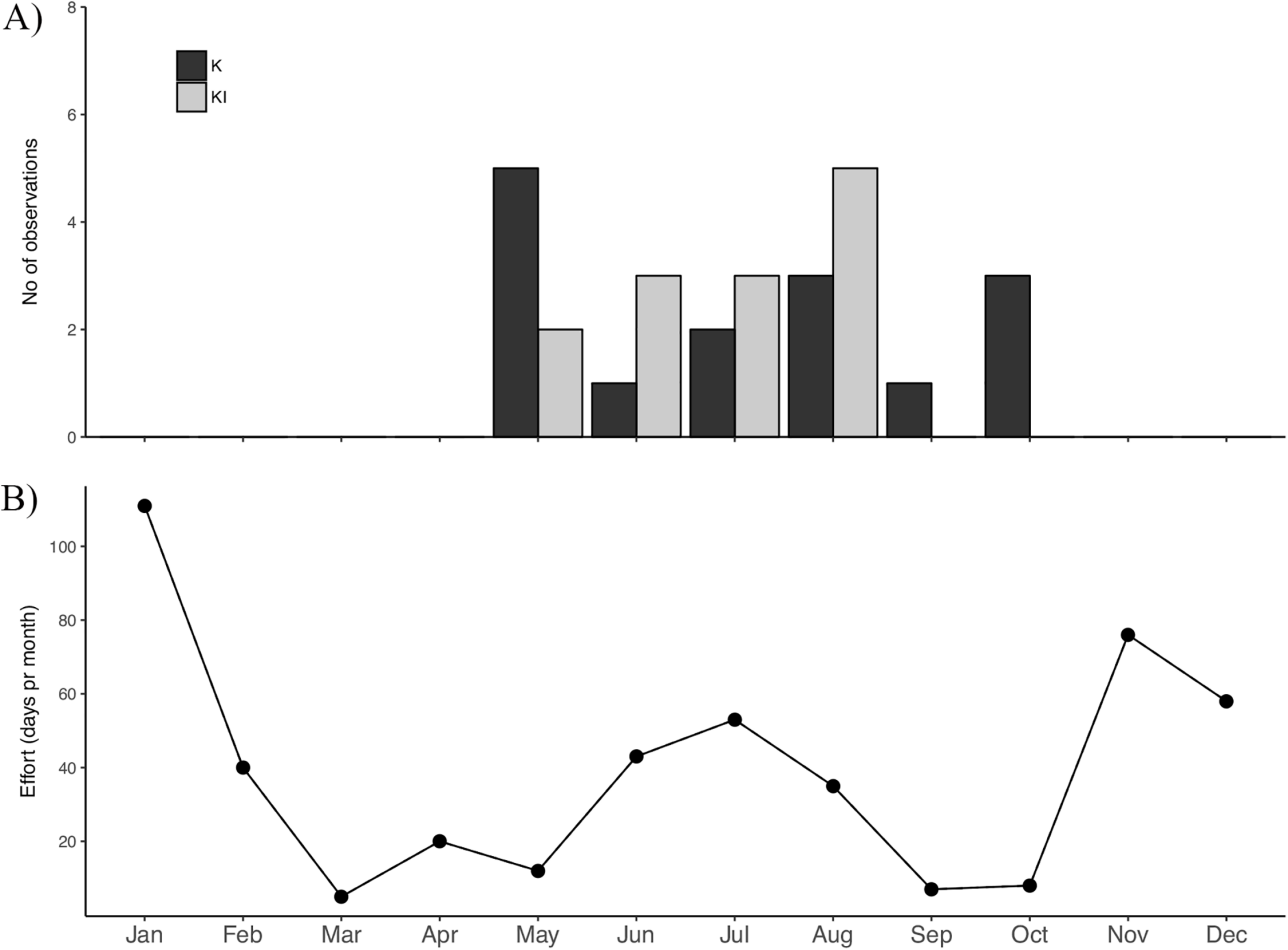
Observations of seal-feeding killer whales in relation to effort from 2010 through 2016. The plot (A) shows the cumulative sighting distribution of KI (dark bars) and K (light bars) whales while the plot (B) reports on the cumulative number of effort days spent in the field for each month, from 2010 through 2016 in northern Norway.

From 1988 through 1992 off the central coast of Norway, KI whales were encountered on six observation days during the months of March (5) and October (1). Between 2010 and 2016 off northern Norway, these whales were observed on 13 days from May through August (Table 2 and Fig 4). K whales were also encountered on 15 occasions between 2010 and 2016, with sightings from May through October (Table 2 and Fig 4). Observations covered a maximum range of 800 km between re-sightings of KI whales and 350 km for K whales.

**Table 2:** Sighting histories of KI and K whales.

Group	1988	1989	1990	1991	1992	2010	2011	2012	2013	2014	2015	2016	Total
KI	1	0	1	3	1	0	1	1	4	2	2	3	19
K	0	0	0	0	0	1	0	0	2	5	3	4	15

The number of encounters with KI and K whales is given for each year during the periods 1988–1992 and 2010–2016.

KI and K whales were not identified on the former herring wintering grounds in Tysfjord, where 584 individual killer whales were identified from 1986 through 2003 (work of Similä and colleagues, mentioned in [36]). They were also not encountered on the recently established herring wintering ground off Troms and Andfjord regions where 656 individuals where identified during November-February and, despite substantial effort, between 2010 and 2016 (Fig 4; [34]).

All encounters with KI and K groups documented in this study occurred within 300 meters from the shoreline or around reefs and seal haul-outs.

### Group size and social organization

Mean group size for seal-feeding killer whales, accounting for both KI and K assemblages, was 5 individuals (N = 27, range = 3–11, median = 5). Fish-feeding killer whale groups encountered during spring-summers 2014, 2015 and 2016 averaged a group size of 13 individuals (N = 30, range = 6–25, median = 12), comparable to findings of Similä and colleagues [27]. The Mann-Whitney-Wilcoxon test supported a statistical difference between group size adopted by seal *vs* fish-feeding killer whales (W = 782.5, p<0.001).

**Fig 5:**
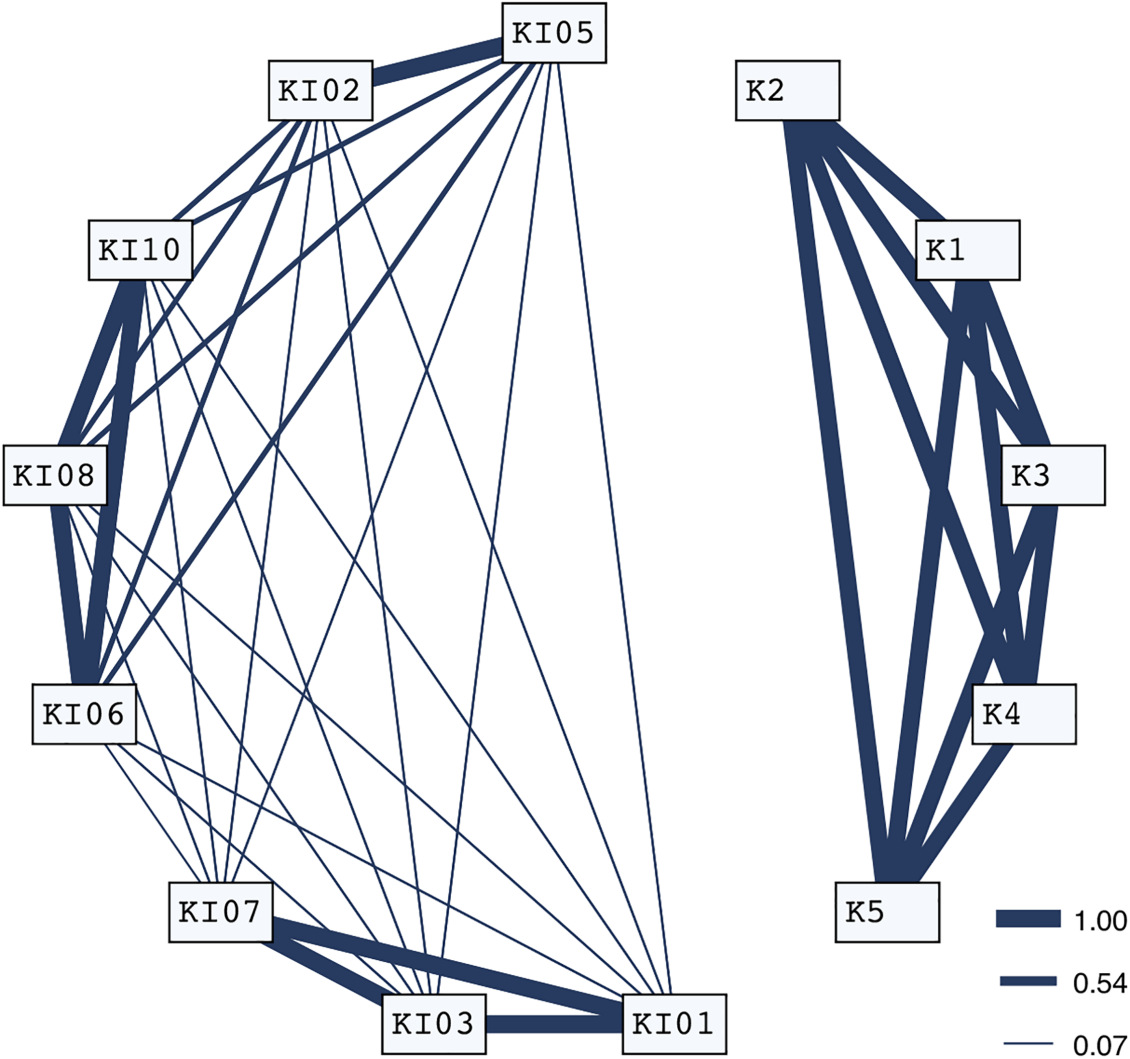
Sociogram of seal-feeding killer whales observed in at least four sampling periods. Occurrence and thickness of links show association patterns between pairs of individuals, revealing the two assemblages KI and K.

**Fig 6:**
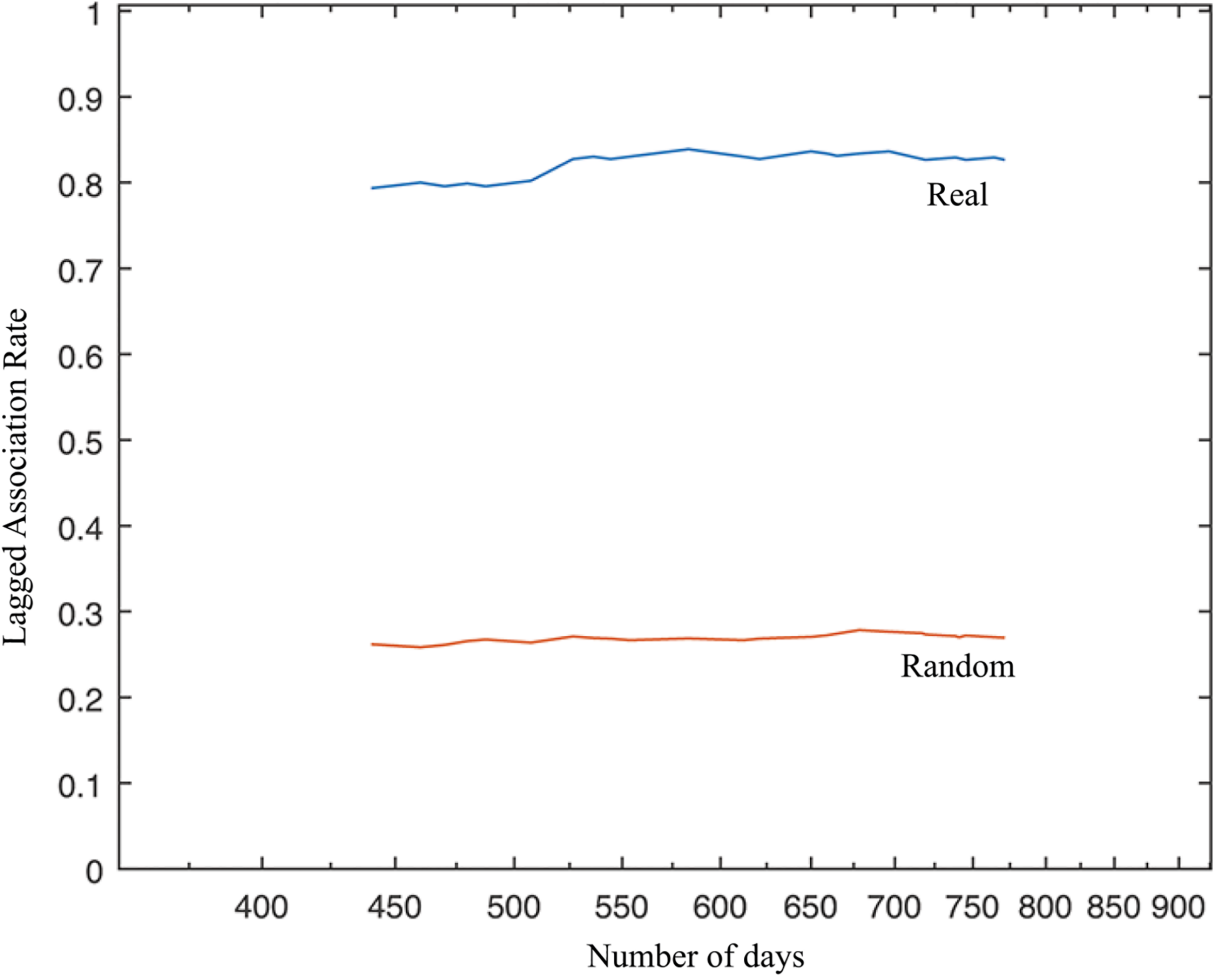
Plots of lagged association rates. The blue plot represents our real data set while the red plot shows the expected lagged association rates if individuals associated at random.

Sixty-eight identifications of 13 individuals over 17 sampling periods (= days) indicated two levels of association. First, individuals associated in 100% of observations enabled four apparently cohesive units to be identified. A second social level, involving temporary associations between cohesive units further indicated two distinct larger assemblages each represented by KI and K whales (Fig 5). The SD of calculated pairwise association indices was significantly higher for the real data set than for the random data (SD_Real_ = 0.40031, SD_Random_ = 0.37726, p = 0.0010), enabling us to reject the null hypothesis that individuals associate at random. Temporal analyses revealed stable associations over long periods of time and above the null association rate (Fig 6).

## Discussion

We have documented a minimum of 13 killer whales, belonging to four stable units, and two larger assemblages (KI and K), that appear to regularly feed on seals in Norwegian coastal waters. Repeated observations over time of identified individuals preying on seals support persistent preference for pinniped prey over several years for K whales, and for nearly three decades for KI-03, KI-05 and KI-06. A comparison of KI and K whales’ behavioral traits to the social structure, occurrence pattern and foraging behavior described for pinniped-feeding killer whales in other regions [9–11, 18, 22, 47, 48] provide further insights into the degree of prey specialization for these whales.

Observations of KI and K whales were widely distributed along the Norwegian coast, with all sightings concentrated in important breeding areas for harbor and grey seals. Major breeding sites for both pinniped species can be found in Froan (64°05’N, 09°15’E), central Norway and in the northern part of the recorded range of this study [49, 50]. With one notable exception discussed below, all encounters with KI and K whales occurred in near shore shallow areas, typical pinniped habitat [49, 50] where the whales were observed following reefs and shoreline contours, or circling haul-outs with long dives, erratic swimming patterns and no real travel direction. This 'haul-out and near shore foraging' behavior has been described for transient killer whales hunting pinnipeds in the Northeastern Pacific [9, 10], at Subantarctic Marion Island and Possession Island, Crozet [47, 48]. Even though seals are non-migratory, thus offering a constant and predictable prey resource throughout their range along the Norwegian coast, it may be more rewarding for killer whales to forage around haul-outs in the breeding season where seal pups and females regularly enter and exit the water. Pacific transient killer whales appear to adapt their range, following latitudinal clines in timing of pupping [10]. Similarly, observations in northern Norway suggested a likelihood for KI and K whales to be observed near haul-outs at times coinciding with pupping periods of harbor seals (June-July, [51]) and grey seals (September-October, [50]) in this region. The timing of observations also suggests a north-south movement pattern for these whales, which coincides with local peaks in pupping and weaning of pinnipeds. More specifically, K whales travelled from the Nordland region where they were observed from May through September to the Troms region where sightings were recorded in late October. This same pattern was repeated in 2014, 2015 and 2016, and observations followed the cline of the grey seal pupping period, which is delayed further north and occurs as late as November-December northwards in the Troms and Finnmark regions [52].

Further evidence supporting prey specialization in KI and K whales is a group size significantly smaller than the group size of herring-feeding killer whales. Living in small groups has been suggested as a strategy to maximize energy intake for pinniped-feeding killer whales. Small group sizes seem to guarantee stealthy and cooperative prey-searching and handling, whilst optimizing prey sharing [11]. In our dataset, small groups of seal-feeding killer whales were also long lasting, as revealed by temporal analyses of social organization. This is consistent with long-term cohesiveness of basic social units found to be a characteristic of the species, regardless of the prey-type hunted (e.g. [8, 22, 37, 53]). Stable and long-lasting social groups undoubtedly provide the raw material needed for social transmission of feeding behaviors and persistence of dietary habits over the years [54]. Additionally, temporary loose associations between stable units may be necessary for social learning of hunting skills and prey handling, and may explain the larger groups that were observed on a few occasions [10, 22]. On 27 June 2015, the three groups of KI whales were observed cooperatively hunting and sharing three seals over a period of six hours. Two juveniles under three years of age and a calf of the year were recorded in the group, and social play was observed when the whales handled a harbor seal pup for 40 minutes before killing it.

Two acoustic recordings available for K whales whilst handling seals at a close distance revealed no audible sounds, but sporadic trains of echolocation clicks from the whales. This is consistent with findings for marine mammal feeding killer whales from other regions [18, 55, 56], and is in sharp contrast to the highly vocal herring-feeding killer whales [57].

KI and K whales seem to have adopted strikingly similar adaptations to other pinniped-feeding killer whales. KI whales were observed feeding on herring in the spawning grounds in 1991 where they were also seen preying on seals. Despite massive study efforts and hundreds of identifications, KI and K groups were not identified on former or current herring wintering grounds during 1986–2003 and 2013–2016, respectively [34]. This could suggest that herring does not represent a main prey resource to these whales. However, because killer whale groups may display variations in habitat usage and preferences, and because data collection so far was largely opportunistic and covering a limited area, individuals might be undetected and not available for sampling in surveyed areas. Thus, absence of KI and K whales from the herring wintering grounds remain inconclusive.

How long these two assemblages have shown this type of prey specialization, and the origins of their distinctive diet remain largely unknown, but some major factors could be at least partially explanatory. The NSS stock of herring, the main prey base for killer whales in Norwegian coastal waters, collapsed in 1970 [58, 59]. How killer whales coped with prey depletion remains unknown, however they would likely have the phenotypic plasticity to adapt to changing environments as suggested by the wide variety of prey items and elaborated feeding techniques documented throughout their range [6, 60, 61]. Whilst prey switching appears as a potential adaptive response to decreasing prey supplies for numerous predator species [62–64], it is possible that some killer whale groups had diversified their diet and developed specific foraging strategies for new prey types in response to the drastic decrease in herring biomass [65]. Vongraven and Bisther [30] discussed this as a potential explanation for the observation of KI whales observed feeding in association with known herring feeding-killer whales in the spawning areas in March 1991, before splitting off into a smaller group and travelling a few miles to inshore waters where they started chasing harbor seals. The past culling of the killer whale population, organized off the central Norwegian coast as a management action to preserve the remaining herring, may also have played a role in the niche diversification process [66, 67]. In 1969 and 1970, 167 and 201 killer whales were culled, respectively, over a 100 x 50 km area and within a few weeks. Entire groups were apparently never culled, and catch records showed a general lack of small individuals and a skewed sex ratio of 2M:1F [67]. Due to differential roles played by key members in cetacean societies and social transmission of matrilineal-based prey specializations in killer whales, feeding strategies may have been lost in culled groups, jeopardizing feeding success if key members were removed, and promoting potential changes in diet [68, 69].

This study revealed two assemblages of killer whales that persistently fed on seal prey and displayed parallel adaptations to that of pinniped-feeding killer whales from other regions, suggesting a certain degree of prey specialization. It remains, however, unknown as to whether KI and K whales are seasonal prey-specialists, switching between different prey types, or if they specialize on mammals year-round. Despite the observations of KI and K whales fitting well with the distribution of pinniped prey over the years, all observations have been recorded within coastal areas covered by the annual migration of the NSS herring. Thus, further analyses of dietary markers in biopsy samples are warranted, and this will constitute a research priority in order to conclude on the degree of prey specialization of these whales.
